# Quantitative and qualitative 3D analysis of mandibular lingual concavities: Implications for dental implant planning in the posterior mandible

**DOI:** 10.1002/cre2.858

**Published:** 2024-02-12

**Authors:** Ahmed Yaseen Alqutaibi, Mohammed Ahmed Alghauli, Afaf Aboalrejal, Abduljabbar K. Mulla, Anwar A. Almohammadi, Abdullah W. Aljayyar, Shahad O. Alharbi, Esam S. Almuzaini, Ahmed K. Alsaeedi, Lina F. Arabi, Mohammed Nasser Alhajj, Esam Halboub

**Affiliations:** ^1^ Substitutive Dental Science Department, College of Dentistry Taibah University Al‐Madinah Saudi Arabia; ^2^ Department of Prosthodontics, Faculty of Dentistry Ibb University Ibb Yemen; ^3^ Oral Biology Department, College of Dentistry Ibb University Ibb Yemen; ^4^ College of Dentistry Taibah University Al Madinah Saudi Arabia; ^5^ Department of Prosthodontics, Faculty of Dentistry Thamar University Dhamar Yemen; ^6^ Department of Maxillofacial Surgery and Diagnostic Sciences, College of Dentistry Jazan University Jazan Saudi Arabia

**Keywords:** CBCT, dental implant, lingual concavity, ridge type

## Abstract

**Objective:**

The purpose of this study is to investigate the type of ridge (degree of angulation of the lingual concavity) and the buccolingual dimensions in the area of the first and second molars in both genders of different ages and how this will affect implant placement in the posterior mandible.

**Materials and Methods:**

This retrospective cross‐sectional study comprised cone beam computed tomography images of 150 dental patients (75 males and 71 aged ≥30). The following were measured/reported: type (morphology) of the ridge (convex [C], parallel [P], or undercut [U]), buccolingual width at the base and the crest of the ridge, and ridge height. The concavity angle, depth, and length of the U‐shaped ridge were measured too.

**Results:**

The prevalence of type U ridge ranged from 32.7% in the first molar region to 62.7% in the second molar region. Almost all measurements and ridge type distributions were comparable amongst the age groups (*p* > .05). Very few significant differences were found when comparing #36 versus #37 and #46 versus #47 teeth, with no differences in the distribution of the ridge types (*p* > .05). Quite the inverse, all measurements were statistically different when comparing #36 versus #37 and #46 versus #47 teeth, and type U ridge was more frequent in second molar compared to the first molar regions, respectively (*p* < .05). Many measurements were statistically higher in females; the inverse was true for a few measurements (*p* < .05). Type U ridge in #36 and #37 was found more frequently among males (*p* < .001). In contrast, the ridge types in #37 and #47 were not statistically different gender‐wise.

**Conclusions:**

The U type of ridge was more prevalent in the investigated population, encountered more frequently in the second molars generally and in the first molars of males than females. Most posterior mandibular measurements are similar age‐ and side‐wise but seem different gender‐ and tooth‐wise.

## 1 INTRODUCTION

Dental implantology is amongst the most sought‐after treatments nowadays in dental practice. When planned carefully, it is associated with high success rates, which means low rates of complications (Misch & Wang, 2008). However, several surgical problems during the dental implant placement may arise, most commonly attributed to prior poor planning. Therefore, the first key to successful dental implant prostheses is a comprehensive diagnosis and a scientifically solid treatment plan (Resnik, 2020).

In this context, many anatomical limitations must be considered before the dental implant surgical procedures to select the proper implantation site, predict and plan implant correct angulation, and avoid peri‐ or postoperative complications or clinical failure (Watanabe et al., 2010). For instance, in the mandibular posterior area, the size, shape, height, and width of the accessible bone and the position of the mandibular canal must be assessed precisely to determine the proper implant size and orientation (Alqutaibi et al., 2022; Datta et al., 2022; Magat, 2020; Tan et al., 2021). Moreover, careful consideration of the lingual concavity, an anatomical variation presented as a slight depression on the medial surface of the mandible above the mylohyoid line, is critical as it may restrict bone availability and increase the risk of surgical problems (Haj Yahya et al., 2021; Herranz‐Aparicio et al., 2016; Kong et al., 2021).

The perforation of the lingual plate during implant placement in the mandibular posterior region is widespread, particularly in cases with deep lingual concavity (Chan et al., 2011; de Souza et al., 2016). Besides, the posterior mandible cross‐sectional shape (referred to herein as “type of the ridge”) also affects lingual plate perforation (Huang et al., 2015; Magat, 2020). It is classified as undercut (U) where the crestal bone width is more than the width in the base; parallel (P), where the width at the crest and base are equal; or convex (C), where the crestal bone width is less than its width as the base. The most frequent complication is associated with type U ridge (Chan et al., 2011). The perforation of the lingual plate may lead to catastrophic complications such as bleeding, inflammation, infection, and life‐threatening conditions such as airway obstruction (Kong et al., 2021; Ramakrishnan, 2020).

The conventional two‐dimensional (2D) imaging approaches have inherent shortages where assessing measurements that need the depth dimension, particularly the buccolingual width, is difficult, if not impossible. Therefore, there is a need for using advanced imaging technology that provides three‐dimensional (3D) visualization of the mandible such as cone beam computed tomography (CBCT) to rid out the above‐inherited shortages of 2D technologies. In fact, CBCT is very effective in detailing the mandibular anatomical structures, such as the submandibular canal, lingual and mental foramina, and associated bony canal changes. Indeed, CBCT is a powerful tool for diagnosis and treatment planning (Riecke et al., 2015; Tang et al., 2017).

A comprehensive understanding of mandibular lingual concavities and their variations is paramount in implant dentistry (Bodart et al., 2020; Nawwar, 2022; Sun et al., 2023; Tan et al., 2021; Yoon et al., 2017). These concavities are crucial in determining the success and longevity of dental implants in the mandibular arch. Knowledge of their location, depth, and types is vital for implantologists to plan implant placement accurately and ensure patient safety and optimal oral health. Failure to account for these concavities can result in improper implant angulation, inadequate prosthetic support, and even damage to vital anatomical structures, such as the inferior alveolar nerve and lingual artery. This study sought to assess, using CBCT, the variations of the mandibular lingual concavities, namely the degree of angulation (referred to herein as the type of the ridge) and the buccolingual dimensions in the first and second molar regions. The between‐groups null hypotheses state “no sex‐ and age‐related differences in the measured outcomes.” The within‐group null hypotheses state “no differences in the measured outcomes between the right and left sides in the mandibular first or second molar regions, and between the first and second molars in each side.”

## 2 MATERIALS AND METHODS

The proposal for this retrospective, cross‐sectional study was approved by the Research Ethics Committee, College of Dentistry, Taibah University (Approval # 12042022). CBCT scans of adult patients who attended Taibah University Dental Hospital (TUDH) between 2018 and 2022 and their demographic and clinical data were retrieved. To be included, the CBCT scan should belong to a patient aged 18 years and have the following criteria: the presence of sound mandibular first and second molars on both sides, no alveolar bone resorption in this investigated area, and no apparent pathology lesions in the apical and periodontal area. Moreover, the CBCT scans should clearly outline the mandible and inferior alveolar canal. On the other hand, CBCT scans were excluded if they had any abnormalities in the area of interest, such as a history of trauma, disease, surgical intervention, congenital anomaly/syndrome, fracture, or any other foreign body.

As a routine practice, CBCT scans followed a standardized scanning protocol. All CBCT scans were taken using the TUDH's CBCT machine (KaVo Dental GmbH), utilizing the following exposure setting: 120 kVp and 5 mA, 16 × 13 cm field of view, 26.9 s acquisition time, and a 0.25 mm voxel size. The study measurements were performed using the RadiAnt DICOM viewer.

To standardize the measurements, the mandible was reconstructed using coronal, sagittal, and axial views. Cross‐sectional sections relevant to the evaluated tooth were obtained in the coronal plane. For this purpose, the coronal section closest to the mesiodistal midpoint of the evaluated tooth was used to determine the cross‐sectional morphology. The inferior alveolar canal, alveolar crest, and mandibular lingual concavity were used as reference points for the analysis in these sections. Throughout the manuscript, #36 represents the left mandibular first molar; #37 is the left mandibular second molar; #46 is the right mandibular first molar; #47 is the right mandibular second molar.

**Figure 1:**
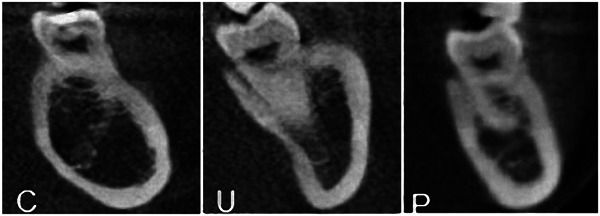
Ridge morphology types: type C (convex), U (undercut), and P (parallel).

**Figure 2:**
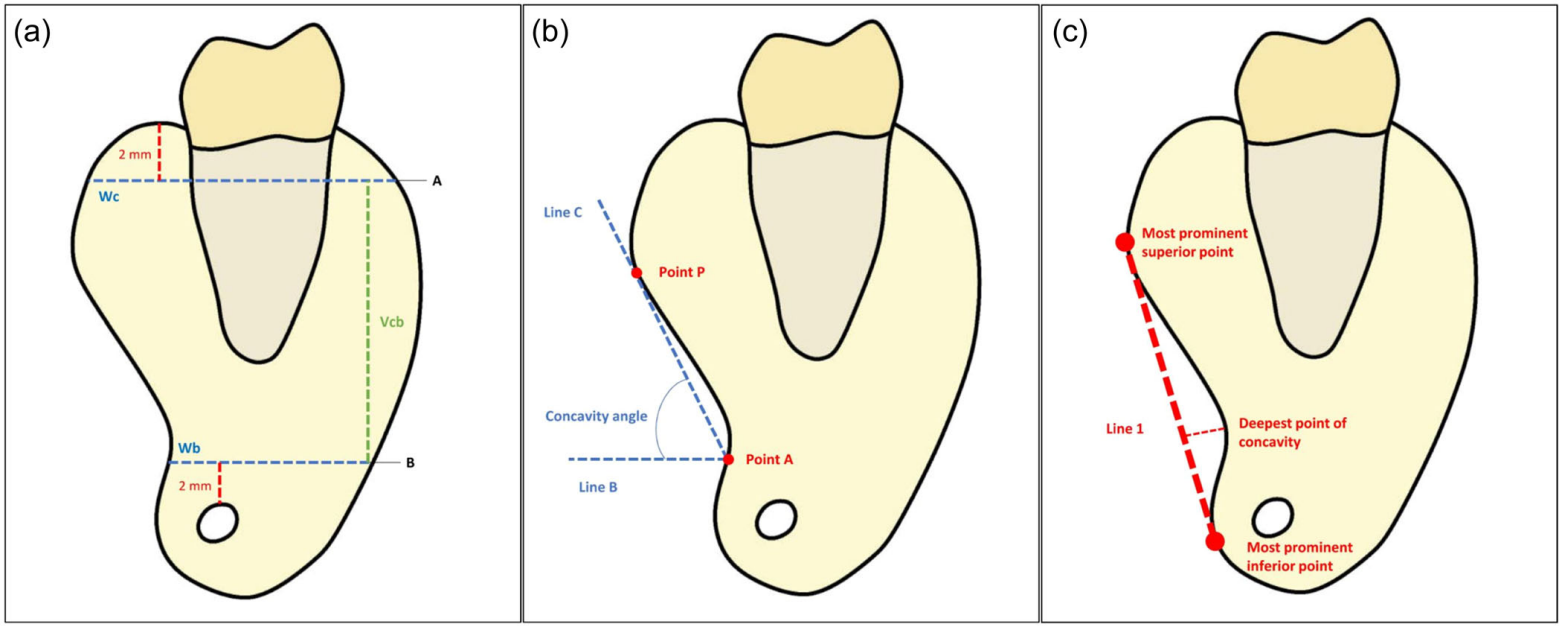
(a) Presentation of alveolar ridges dimensional measurements (Wc, 2 mm below the alveolar ridge crest buccolingual width of ridges; Wb, 2 mm above inferior alveolar canal buccolingual width of ridges; Vcb, the height of the alveolar ridge). (b) Presentation of concavity angle measurements of U‐shaped ridges, and (c) presentation of concavity depth and length measurements of U‐shaped ridges.

The ridge was categorized into U, C, or P based on Chan et al. criteria (2011) as shown in Figure 1. Type U ridge was decided if the crest was wider than the base. The opposite is true for type C. While type P ridge is represented by an equal crest and base. As indicated in Figure 2a, a horizontal line (named B) was drawn 2 mm above the superior border of the inferior alveolar canal. “Wb” represented the ridge's buccolingual width at the level of line B. Another horizontal line (named A) was drawn 2 mm below the highest point in the lingual plate. “Wc” represented the ridge's buccolingual width at the level of line A. The height of the alveolar ridge (Vcb) was measured as the dimension of the perpendicular line joining the horizontal lines A and B.

Given that the type U ridge has the highest risk of lingual plate perforation, the degree of lingual concavity was assessed therein. In this context, the following variables were measured for the type U ridge only: the concavity angle, the concavity length, and the concavity depth. As shown in Figure 2b, the angle B‐A‐C was used to measure and describe the lingual concavity of the alveolar bone (i.e., concavity angle) where point A, represents the deepest point of lingual concavity. Point P represents the most prominent superior point of lingual concavity. A line named B is a horizontal line that passes through point A, and a line, named C, passes from point A in a superior direction and touches the lingual plate in point P tangentially.

As demonstrated in Figure 2c, the most prominent point above the lingual concavity is marked and called “the most prominent superior point.” Similarly, the most prominent point below the lingual concavity is marked and called “the most prominent inferior point.” A line that tangents these two points (without passing through the lingual plate) is called line 1. The line drawn perpendicular to line 1 and extended to point A of the concavity was measured as the depth of the lingual concavity.

**Figure 3:**
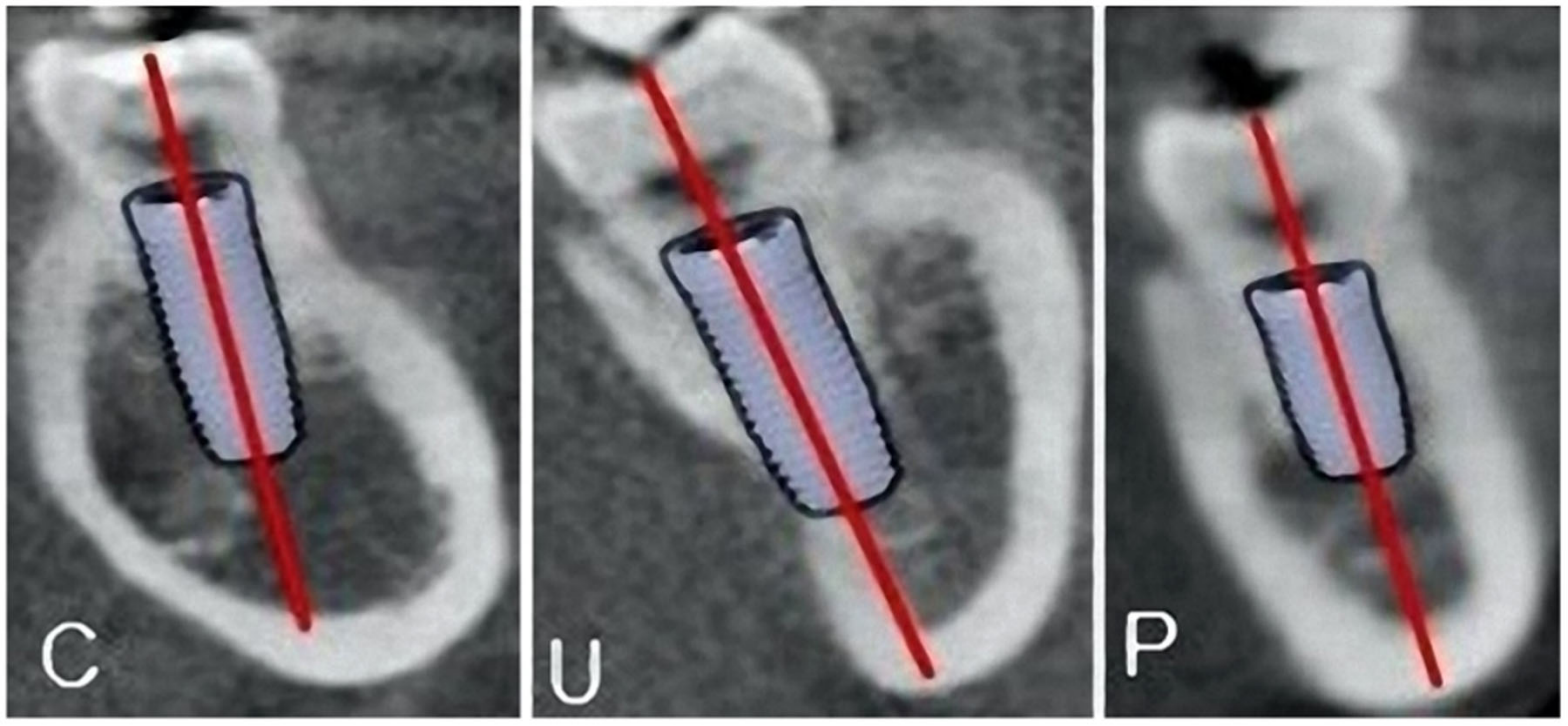
Virtual implant placement in three ridge types: type C (convex), U (undercut), and P (parallel).

Figure 3 shows the virtual implant placement in three types of ridges and the risk of lingual plate perforation associated with type U ridge.

Two calibrated and trained examiners conducted all measurements independently on two different occasions at a 1‐month interval. Intraclass correlation (ICC) was conducted to assess the measurements' reliability (intraobserver and interobservers). Besides the high reliability achieved, the adopted measurements were the averages of the two readings of the individual examiners.

The collected data were tabulated based on patients' age, gender, tooth type, and the side of the mandible to be ready for statistical analysis. All statistical analyses were performed using SPSS version 28 (IBM Corp.). The normal distribution of data was assessed by the Shapiro–Wilk test. The differences in the mean values of the CBCT measurements between the age groups (18–29 vs. ≥30 years) and gender were evaluated using the independent *t*‐test. *χ* ^2^ test was used to see whether there may be an association between the different types of ridge morphology and the types of tooth. The paired *t*‐test was used to compare the measurements tooth‐ and side‐wise. Where the variables of the degree of the lingual cavity were considered (the concavity angle, the concavity length, and the concavity depth), only the type U concordances were included in the comparisons. Accordingly, the sample sizes were different wherever these variables were compared either between groups or within groups. McNamara test was conducted to assess the distribution of ridge types tooth‐ and side‐wise. Regarding the classification of the ridge type, intra‐ and interrater reliability was done using Cohen's *ϰ* coefficient.

### 2.1 Ethics statement

The research protocol received approval from the Faculty of Dentistry ethics committee at Taibah University, with the reference number #12042022 and each patient voluntarily provided written consent after being fully informed.

## 3 RESULTS

Out of 1098 screened CBCT radiographs, 150 scans were selected as they fulfilled the inclusion criteria. The sample comprised 150 participants; exactly half of them (50%) were males. Seventy‐nine patients (52.6%) aged between 18 and 29 years, and 71 patients (47.4%) aged ≥30 years. Gender‐wise, the age distribution was comparable (*p* > .05). ICC revealed very high intra‐ and interrater agreements ranging from as low as 0.92 to as high as 1. Similarly, Cohen's *ϰ* coefficient ranged from as low as 0.96 for #46 to as high as 1 for #37 and #47.

The results of all measured outcomes for each molar for the whole sample and by gender and age groups are shown in Tables 1 and 2. All measurements were comparable in these two age groups (*p* > .05), except for two measurements of tooth #37: The “height of the ridge” was significantly higher for age group ≥30 (11.45 ± 2.73 vs. 10.58 ± 2.61 mm; *p* = .012), while the “crest width” was significantly lower (13.45 ± 2.52 vs. 14.53 ± 2.67 mm; *p* = .049).

**Table 1:** Study measurements of all and by gender and age group for left first and second molars.

Tooth	Variable	ALL (*N* = 150)	Age groups				Gender			
			18–29 years	≥30 years	*n*	*p*	Males	Females	*n*	*p*
#36	Base width	12.0 ± 2.6	12.0 ± 2.6	11.9 ± 2.6	150	.774	10.8 ± 2.3	13.2 ± 1.4	150	**<.001**
	Crest width	11.4 ± 1.2	11.5 ± 1.3	11.3 ± 1.2	150	.369	11.3 ± 1.1	11.5 ± 1.4	150	.416
	Ridge height	13.0 ± 2.8	12.8 ± 2.3	13.2 ± 3.3	150	.376	13.8 ± 3.1	12.2 ± 2.2	150	**<.001**
	Concavity angle	51.5 ± 12.4	44.9 ± 12.3	54.0 ± 12.4	52	.250	37.6 ± 13.7	44.1 ± 9.9	88	.750
	Concavity length	11.5 ± 2.5	11.4 ± 2.6	11.6 ± 2.4	52	.844	10.8 ± 3.7	12.9 ± 2.0	88	**.001**
	Concavity depth	2.0 ± 0.8	1.9 ± 0.7	2.1 ± 0.8	52	.397	1.8 ± 0.8	1.9 ± 0.7	88	.477
#37	Base width	13.0 ± 2.7	13.2 ± 2.6	12.8 ± 2.9	150	.370	11.9 ±± 2.5	14.2 ± 2.4	150	**<.001**
	Crest width	14.0 ± 2.7	14.5 ± 2.7	13.5 ± 2.5	150	.012	12.7 ± 1.6	15.4 ± 2.8	150	**<.001**
	Ridge height	11 ± 2.69	10.6 ± 2.6	11.5 ± 2.7	150	.049	11.5 ± 2.9	10.5 ± 2.4	150	**.020**
	Concavity angle	43.8 ± 9.2	42.9 ± 8.5	44.8 ± 10.0	94	.337	44.6 ± 10.2	42.9 ± 8.1	94	.384
	Concavity length	12.5 ± 2.2	12.7 ± 2.1	12.3 ± 2.3	94	.415	12.0 ± 2.3	13.0 ± 1.9	94	**.028**
	Concavity depth	2.1 ± 0.8	2.0 ± 0.8	2.2 ± 0.8	94	.149	2.2 ± 0.9	1.9 ± 0.7	94	.095

*Note*: Bold values are statistically significant *p* < .05.

**Table 2:** Study measurements of all and by gender and age group for right first and second molars.

Tooth	Variable	ALL (*N* = 150)	Age groups				Gender			
			18–29 years	≥30 years	*n*	*p*	Males	Females	*n*	*p*
#46	Base width	12.2 ± 2.3	12.4 ± 2.4	11.9 ± 2.2	150	.138	11.4 ± 2.2	12.9 ± 2.2	150	**<.001**
	Crest width	11.3 ± 1.1	11.4 ± 1.2	11.2 ± 1.1	150	.274	11.2 ± 0.9	11.4 ± 1.3	150	.474
	Ridge height	13.2 ± 2.4	12.8 ± 2.3	13.5 ± 2.4	150	.088	13.5 ± 2.4	12.8 ± 2.3	150	.107
	Concavity angle	52.7 ± 15.8	52.3 ± 17.3	53.0 ± 14.9	49	.876	50.0 ± 15.9	60.1 ± 13.6	49	**.048**
	Concavity length	10.6 ± 3.0	10.9 ± 3.5	10.4 ± 2.5	49	.574	10.6 ± 3.1	10.7 ± 2.7	49	.949
	Concavity depth	1.7 ± 0.8	1.7 ± 0.9	1.7 ± 0.6	49	.865	1.8 ± 0.8	1.6 ± 0.9	49	.456
#47	Base width	13.2 ± 2.6	13.5 ± 2.5	13.0 ± 2.7	150	.112	12.5 ± 2.6	14.0 ± 2.4	150	**<.001**
	Crest width	13.7 ± 2.3	14.1 ± 2.3	13.1 ± 2.2	150	.274	12.7 ± 1.6	14.6 ± 2.5	150	**<.001**
	Ridge height	11.1 ± 2.5	10.75 ± 2.4	11.4 ± 2.6	150	.091	11.5 ± 2.6	10.7 ± 2.4	150	**.043**
	Concavity angle	43.8 ± 9.9	46.4 ± 18.0	52.3 ± 17.7	80	.075	49.0 ± 17.8	49.1 ± 18.4	80	.494
	Concavity length	12.3 ± 2.2	10.0 ± 3.3	9.9 ± 3.4	80	.968	10.3 ± 3.6	9.6 ± 3.1	80	.327
	Concavity depth	2.1 ± 0.7	1.6 ± 0.9	1.5 ± 0.9	80	.677	1.7 ± 1.0	1.4 ± 0.9	80	.089

*Note*: Bold values are statistically significant *p* < .05.

Regarding the gender‐wise results, the following measurements were statistically higher in females compared to males: base width of #36 (13.21 ± 1.39 vs. 10.75 ± 2.27; *p* < .001), base width of 37 (14.18 ± 2.39 vs. 11.86 ± 2.51; *p* < .001), crest width of 37 (15.36 ± 2.82 vs. 12.67 ± 1.56; *p* < .001), height of the alveolar ridge of #37 (12.84 ± 1.91 vs. 11.49 ± 2.95, *p* = .020), concavity length of #36 (12.9 ± 2.03 vs. 10.87 ± 3.68, *p* = .001), concavity length of #37 (12.97 ± 1.9 vs. 12 ± 2.32; *p* = .028), base width of 46 (12.94 ± 2.15 vs. 11.41 ± 2.23; *p* < .001), base width (13.95 ± 2.44 vs. 12.53 ± 2.57; *p* < .001), and crest width of #47 (14.62 ± 2.52 vs. 12.67 ± 1.59; *p* < .001). Meanwhile, the following measurements were statistically significantly lower in females compared to males: height of the alveolar ridge of #36 (12.2 ± 2.23 vs. 13.8 ± 3.06; *p* < .001), and height of the alveolar ridge of #47 (10.67 ± 2.36 vs. 11.48 ± 2.56; *p* = .043).

Likewise, no significant differences were found upon comparing the measurements for teeth #36 versus #46, and #37 versus 47 (*p* > .05), except for the crest of the ridge, which was significantly wider at the left second molar (14.02 ± 2.65) compared to the right second molar (13.65 ± 2.32; *p* = .018). The crest width and the base width were higher, while the height of the alveolar ridge was lower in the second molars than in the first molars, irrespective of the side (Table 3).

**Table 3:** Study measurements by tooth and side.

Tooth type	Variable	Side		*p* Value*
		Right	Left	
Dimensions of all ridge types by tooth and side				
First molar (*n* = 300)	Base width	12.2 ± 2.3	12.0 ± 2.6	.172
	Crest width	11.3 ± 1.1	11.4 ± 1.2	.214
	Height	13.2 ± 2.4	13.0 ± 2.8	.426
Second molar (*n* = 300)	Base width	13.2 ± 2.6	13.0 ± 2.7	.227
	Crest width	13.7 ± 2.3	14.0 ± 2.6	**.018**
	Height	11.1 ± 2.5	11.0 ± 2.7	.611
Measurements for type U ridge by tooth and side				
First molar (*n* = 34)	Concavity angle	52.0 ± 16.3	52.7 ± 12.8	.772
	Concavity length	10.8 ± 3.4	12.1 ± 2.6	**.010**
	Concavity depth	1.8 ± 0.8	2.1 ± 0.8	**.011**
Second molar (*n* = 61)	Concavity angle	43.5 ± 8.9	44.1 ± 8.8	.551
	Concavity length	12.6 ± 2.2	12.6 ± 2.3	.842
	Concavity depth	2.2 ± 0.7	2.0 ± 0.8	**.028**

*Note*: Bold values are statistically significant *p* < .05.

Regarding the outcomes of the “degree of the lingual cavity” (the concavity angle, the concavity length, and the concavity depth), only CBCT where concordances in type U ridges existed were included according to the different comparisons (#36 vs. #37, 46 vs. #47, #36 vs. #46, and #37 vs. #47). The concavity angle was higher in #36 and #46 (51.34 ± 34 and 52.34 ± 16.11, respectively) compared to #37 and #47 (43.13 ± 10.31 and 45.47 ± 11.23, respectively, *p* < .001 and .015). Concavity length in #46 was statistically lower than that of #36 and #47 (*p* = .010 and .005, respectively). The concavity depth was significantly higher in #47 compared to #46 (*p* = .006), in #47 compared to #37 (*p* = .028), and #36 compared to #46 (*p* = .011). More details are presented in Table 3.

**Figure 4:**
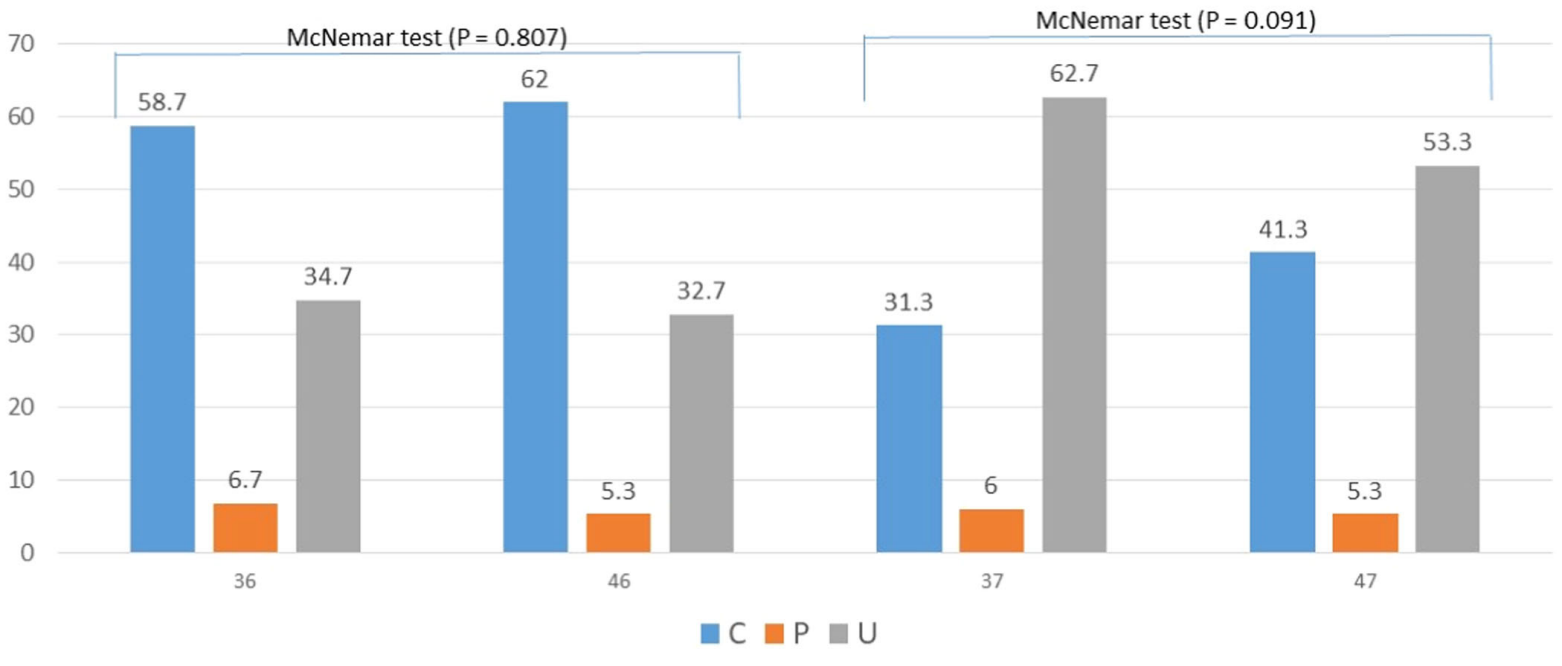
The ridge type distributions of the ridge types in #36 versus #46 and #37 versus #47.

**Figure 5:**
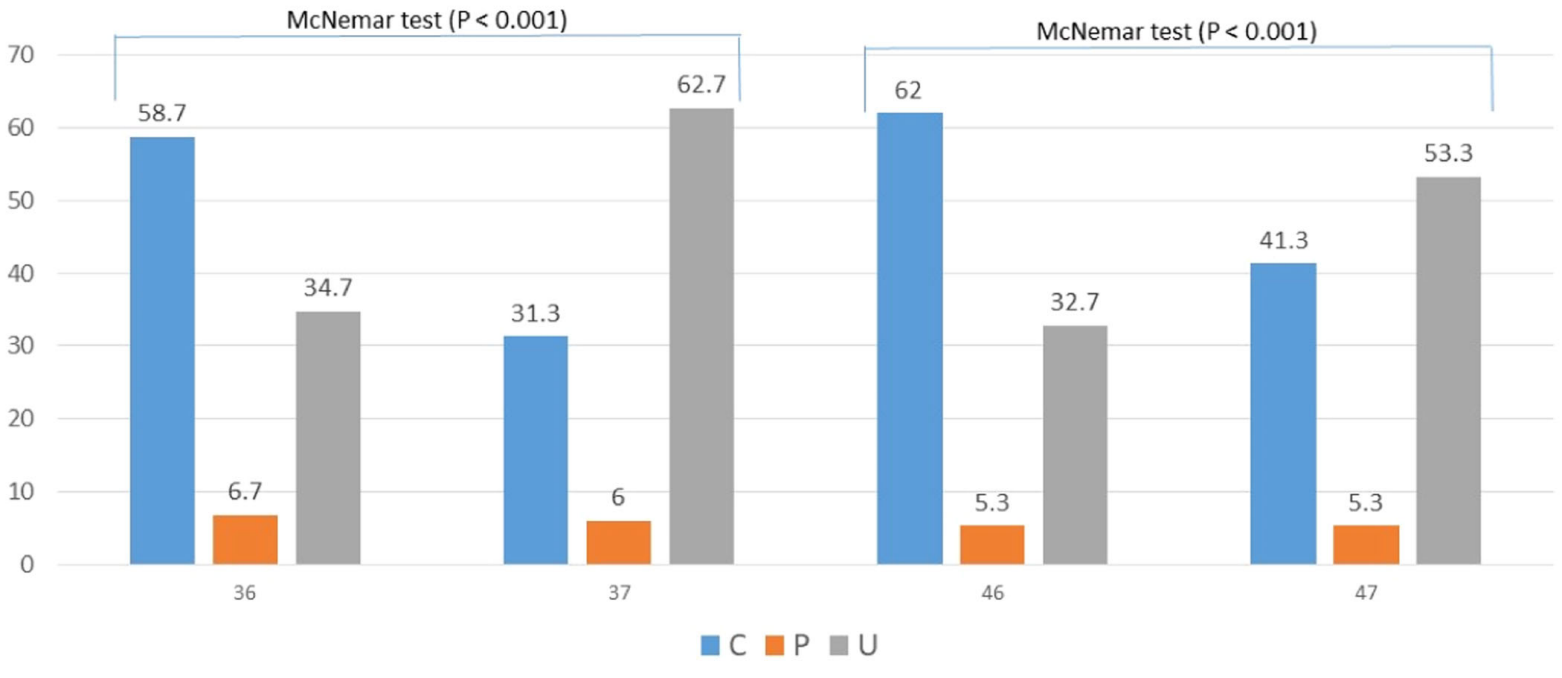
The ridge type distributions in 36 versus 37 and 46 versus 47.

The ridge types of #36 and #46 were statistically different by gender: most of the fractions of types C and P were found among females (64% and 60%, respectively, in #36, and 60% and 75%, respectively, in #46), while most of the fraction of type U was found among males (77% in 36 and 74% in 46; *p* < .001 each). In contrast, the ridge types in #37 and #47 were not statistically different gender‐wise (Table 4). The distributions of the ridge types in #36 versus #46 and in #37 versus #47 were almost similar. Contrastingly, the distributions of the ridge types in #36 versus #37 and in #46 versus #47 were statistically different: type U ridge was statistically more frequent in the mandibular second molars compared to the mandibular first molars as shown in Figures 4 and 5.

**Table 4:** Distribution of ridge type by tooth and gender.

Tooth	Ridge type	ALL	Males	Females	*p* Value
#36	Convex	88 (58.7)	31 (35.2)	57 (64.8)	**<.001**
	Parallel	10 (6.6)	4 (40)	6 (60)	
	Undercut	52 (34.6)	40 (76.9)	12 (23.1)	
#46	Convex	93 (62.0)	37 (39.8)	56 (60.2)	**<.001**
	Parallel	8 (5.3)	2 (25)	6 (75)	
	Undercut	49 (32.0)	36 (73.5)	13 (26.5)	
#37	Convex	47 (31.3)	25 (53.2)	22 (46.8)	.841
	Parallel	9 (6.0)	4 (44.4)	5 (55.6)	
	Undercut	94 (62.6)	46 (48.9)	48 (51.1)	
#47	Convex	62 (41.3)	35 (56.5)	27 (43.5)	.249
	Parallel	8 (10.7)	5 (62.5)	3 (37.5)	
	Undercut	80 (53.3)	35 (43.8)	45 (56.3)	

*Note*: Bold values are statistically significant *p* < .05.

## 4 DISCUSSION

The degree of angulation of the lingual concavity (the ridge type), and the buccolingual dimensions in the mandibular first and second molars regions are pivotal for many surgical procedures, particularly dental implantology. Availability, along with knowledge and awareness, of the measurements of the mandibular lingual concavity and other dimensional outcomes of the posterior mandible will assist the oral surgeon in taking the necessary precautions before starting the treatment.

An insight into the averages of the outcomes in our study in comparison to the averages of the same outcomes in a new recent study (Tan et al., 2021) conducted by Tan et al., revealed approximately similar values except for the height of the alveolar ridge where it was higher in Tan et al., study, and except for concavity angle and concavity length, and to a very lesser extent the concavity depth: The former two were higher in Tan et al. study for all molars except the mandibular right second molar where the opposite is true. The differences in the outcomes of the degree of the lingual cavity (the concavity angle, the concavity depth, and the concavity length) are logical because the type U ridge is more frequent in our study (46% of all sites) compared to the Tan et al. (2021) study (33%). But the difference in the height of the alveolar ridge in favor of Tan et al. (2021) study is interesting; it might be attributed to ethnic influence. Based on the results of the current study, the null hypotheses were entirely or partially rejected. Accordingly, the following results on the study population must be considered carefully and thoughtfully and must be translated into health policy actions by informing the relevant dental practitioners and authorities: The higher rate of type U ridges (especially in the second molars regions), the higher averages of many of the outcomes in favor of females compared to the higher averages of only a few other outcomes in favor of males, the differential differences in the averages of many outcomes between first and second molars irrespective of the side, and the approximate lack of differences between each molar with the corresponding molar on the other side.

Gender‐wise differences were interesting. Although the body size of males outweighs that of females, this was not the case in our study in all statistically different outcomes except for the ridge height of the second molars, which were statistically higher among males (Al Sheikh et al., 2019; Kamburoğlu et al., 2015; Tan et al., 2021). It is difficult to explain such gender‐wise differences. Indeed, no absolute agreement yet on this issue in the published literature. Tan et al. (2021) reported statistically higher length and depth of lingual concavities among males than among females, although they reported the opposite regarding the base and crest width and the height of the ridge. Meanwhile, other studies (Kamburoğlu et al., 2015; Larrabee & Bevans, 2016; Nummikoski et al., 1988) reported higher values among males than among females regarding all outcomes. In their turn, Yoon et al. (2017) did not report statistically significant differences in the mandibular lingual concavity neither race‐wise nor gender‐wise.

Almost all outcomes were similar irrespective of age: 18–29 or ≥30 years. The width of the crest and the height of the alveolar ridge at the second left molar area were exceptions: The former was statistically significantly higher among the younger age group, while the latter was statistically higher among the older age group. These results were supported by Kamburoğlu et al. (2015) and Tan et al. (2021). However, these authors reported statistically lower measurements in the older age group. Interestingly, the alveolar ridge height for the elderly was statistically higher than the young group at the mandibular left second molar regions. It is a matter that might not approve the expectation of age‐related alveolar bone loss (Streckfus et al., 1999). Another study reported a negative correlation between age and the height of the mandibular bone (Al Sheikh et al., 2019). One reason for such a difference is that the ages of most of the included patients were near 30 years old, the cut‐point of dividing the patients into two age groups. As the current study was not prospective—the design that follows participants over time to record changes, if any—a sound conclusion cannot be drawn. Moreover, apart from the fact that there was a statistical difference, the difference was too small to be considered clinically significant (0.85 mm).

As expected, there are no side‐wise differences in all outcomes for each individual molar except for the crest width (wider on the left side), the concavity length, and the concavity depth (higher on the left side) at the first molar region. Inversely, the concavity depth was higher on the right side of the second molar region. Surprisingly, Tan et al. (2021) reported statistically significant differences in all measurements on both sides. In contrast, Kamburoğlu et al. (2015) reported a difference in the lingual concavity only between the left and right sides of the mandible.

The differences were many and obvious when the first molar was compared to the second molar on each side. While the concavity angle and height of the alveolar ridge were higher in the first molar region compared to the second molar region on each side, the other outcomes were lower. This is logical to a large extent in light of the results that the prevalence of type U ridges in the second molar region is nearly twice that in the first molar region. Indeed, the higher the value of the concavity angle, the less the prevalence of the type U ridge, the longer the concavity length, and the shallower the concavity depth. In our study, the former notion applies to the concavity angle and the concavity depth but not the concavity length. In support of our results, Tan et al. (2021) and Nickenig et al. (2015) reported that lingual concavity was more prevalent in the second molar region (90%) than in the first molar region (56%).

The most prevalent ridge types in the present study are type C in the first molar region and type U in the second molar region, a matter that necessitates paying emphasis and special care while placing dental implants in the second molar region. Huang et al. (2015) and Sun et al. (2023) supported such findings. Exactly the opposite, the most prevalent ridge type in Tan et al. (2021) and Watanabe et al. (2010) studies was type C in the first and second molar areas. In contrast, in Chan et al. study (2011) the prevalence rate of type C, U, and P ridges were 13.6%, 20.4%, and 66%, respectively.

In agreement with the current study finding, a recent study evaluated mandibular first and second molar areas in a Southeast Asian population. The results showed that second molars presented greater anatomical difficulties for immediate implant placement, including inadequate interradicular bone thickness, a higher incidence of unfavorable mandible shape, and increased proximity to vital structures. The study highlights the importance of considering these factors when planning immediate implant placement in the mandibular molar region (Ho et al., 2022).

CBCT was utilized in our study to evaluate the anatomic variations in the posterior region of the mandible. Indeed, the CBCT image quality and lower dosage and expense compared to traditional computed tomography have made 3D craniofacial assessment easier in dental practice (Suomalainen et al., 2008). Basically, the CBCT parameters affect the image resolution. Hence, all CBCT scans in this study were obtained on the condition that they were taken using the same machine and the same parameters in order not to affect the visibility of the anatomical structures (Jasa et al., 2017). Thanks to technology, CBCT provides a high potential for evaluating other maxillofacial regions in all planes, including the dimensions, location, and prevalence of the lingual concavity, along with the necessary bone quality and quantity before dental implantation. The availability of CBCT estimation of these anatomical regions on different populations will help the operators to tailor appropriate treatment planning, which helps in developing proper implant placement protocols and hence successful implant placement.

Oral surgeons should be aware of the high incidence of lingual plate perforation upon operating on type U ridges (Chan et al., 2011; Herranz‐Aparicio et al., 2016; Huang et al., 2015). Assuming this happens, the extruded implant may cause chronic irritation or infection, with a potential of infection dissemination into the parapharyngeal and retropharyngeal areas, causing catastrophic health scenarios like mediastinitis, mycotic aneurysm formation with internal carotid artery rupture, internal jugular vein thrombosis with septic pulmonary embolism, or upper airway obstruction. Such serious complications must be considered seriously during treatment planning and implantation of the posterior mandible (Ramakrishnan, 2020).

The applicability of the current study's results to different populations or environments might be constrained because the study was conducted exclusively at one location. Nevertheless, it is worth mentioning that Madina, the chosen city, is home to a diverse population encompassing individuals from different racial backgrounds. Moreover, the study solely concentrated on examining the condition of healthy teeth and did not take into account potential variations in outcomes for teeth with dental restorations. Moreover, this study was conducted with a relatively small sample size and most included patients were in their third decade of life. Further, the study included dentate subjects only. Accordingly, we suggest conducting future studies involving a larger sample size from multiple centers and recruiting dentate and edentulous subjects with a wider age range.

## 5 CONCLUSIONS

Within the limitations of this study, the following could be concluded:

1. Lingual concavity is a common finding in the second molar region, specifically the undercut type.
2. The lingual concavity seems to be not affected by age.
3. Most measurements relevant to lingual concavity are higher among females.
4. Tooth‐wise, almost all the measured outcomes are similar.

## AUTHOR CONTRIBUTIONS

**Ahmed Yaseen Alqutaibi**: Conceptualization, methodology, investigation, results, writing—original draft, writing—review and editing, project administration, supervision. **Mohammed Ahmed Alghauli and Afaf Aboalrejal**: Conceptualization, methodology, investigation, results, writing—original draft, writing—review and editing. **Abduljabbar K. Mulla, Anwar A. Almohammadi, and Abdullah W. Aljayyar**: Results, writing—original draft, writing—review, supervision. **Shahad O. Alharbi, Esam S. Almuzaini, Ahmed K. Alsaeedi, and Lina F. Arabi**: Methodology, investigation, results. **Mohammed Nasser Alhajj and Esam Halboub**: Investigation, results, project administration.

## CONFLICT OF INTEREST STATEMENT

The authors declare no conflict of interest.

## Data Availability

The data sets utilized in this investigation can be obtained from the corresponding author upon a reasonable request.
